# Do biological control agents adapt to local pest genotypes? A multiyear test across geographic scales

**DOI:** 10.1111/eva.13682

**Published:** 2024-04-11

**Authors:** Amanda Kyle Gibson, Fabiane M. Mundim, Abbey L. Ramirez, Patricia Timper

**Affiliations:** ^1^ Department of Biology University of Virginia Charlottesville Virginia USA; ^2^ Department of Biology Utah State University Logan Utah USA; ^3^ United States Department of Agriculture Agricultural Research Service Tifton Georgia USA

**Keywords:** biological control, coevolution, genotype‐by‐genotype specificity, local adaptation, *Meloidogyne*, *Pasteuria*, time‐shift experiments

## Abstract

Parasite local adaptation has been a major focus of (co)evolutionary research on host–parasite interactions. Studies of wild host–parasite systems frequently find that parasites paired with local, sympatric host genotypes perform better than parasites paired with allopatric host genotypes. In contrast, there are few such tests in biological control systems to establish whether biological control parasites commonly perform better on sympatric pest genotypes. This knowledge gap prevents the optimal design of biological control programs: strong local adaptation could argue for the use of sympatric parasites to achieve consistent pest control. To address this gap, we tested for local adaptation of the biological control bacterium *Pasteuria penetrans* to the root‐knot nematode *Meloidogyne arenaria*, a global threat to a wide range of crops. We measured the probability and intensity of *P. penetrans* infection on sympatric and allopatric *M. arenaria* over the course of 4 years. Our design accounted for variation in adaptation across scales by conducting tests within and across fields, and we isolated the signature of parasite adaptation by comparing parasites collected over the course of the growing season. Our results are largely inconsistent with local adaptation of *P. penetrans* to *M. arenaria*: in 3 of 4 years, parasites performed similarly well in sympatric and allopatric combinations. In 1 year, however, infection probability was 28% higher for parasites paired with hosts from their sympatric plot, relative to parasites paired with hosts from other plots within the same field. These mixed results argue for population genetic data to characterize the scale of gene flow and genetic divergence in this system. Overall, our findings do not provide strong support for using *P. penetrans* from local fields to enhance biological control of *Meloidogyne*.

## 1 INTRODUCTION

Obligate parasites are under strong selection to infect locally common host genotypes. We expect this selection to result in parasites that are locally adapted if there is both specificity, such that parasite genotypes vary in which subset of hosts they can infect, and genetic divergence of hosts between sites, such that different host genotypes are locally common (Gandon, 2002; Kawecki & Ebert, 2004; Shykoff & Schmid‐Hempel, 1991). Local adaptation means that the average fitness of parasites paired with hosts from their local site (in sympatry) is higher than the average fitness of parasites paired with hosts from foreign sites (in allopatry) (Blanquart et al., 2013; Lively et al., 2004). There are now many tests of local adaptation in wild host–parasite systems, and they find that parasites are frequently, but not universally, well‐adapted to their local hosts (Greischar & Koskella, 2007; Hoeksema & Forde, 2008; Lajeunesse & Forbes, 2002).

Whether biological control agents similarly adapt to locally common pest genotypes remains an open question. This is an important management consideration (Eilenberg et al., 2001). Biological control programs could leverage information on local adaptation to increase the rate and consistency with which natural enemies establish and spread following release. Notably, strong local adaptation might argue for the use of local enemy populations, rather than a standard source population, to promote establishment in a particular field or region (Hufbauer & Roderick, 2005).

In spite of these management implications, we have limited data on adaptation of biological control agents to local host genotypes. Hufbauer (2001) found no evidence that parasitoid wasps (*Aphidius ervi*) were better able to parasitize pea aphids (*Acyrthosiphon pisum*) from their same field. In two larger‐scale studies, reciprocal infections between geographically distant hosts suggest that biological control agents are most effective on hosts from the same region (Benoist et al., 2020; Goolsby et al., 2006). For example, Goolsby et al. (2006) found that *Floracarus perrepae* mites more aggressively attacked lineages of the invasive fern *Lygodium microphyllum* that originated from the same region of Australia. While suggestive, these latter studies compared two geographic areas; more sites are required to conclusively demonstrate local adaptation and estimate its strength (Blanquart et al., 2013). Overall, this body of work supports the potential for parasite local adaptation in biological control systems, but we do not yet have conclusive evidence that it occurs.

In light of this gap, we estimated local adaptation of the biological control parasite *Pasteuria penetrans* to its pest host, the root‐knot nematode *Meloidogyne arenaria*. Root‐knot nematodes (*Meloidogyne* spp.) threaten most crops, including cassava, soybean, cotton, rice, and peanut (Onkendi et al., 2014). They are recognized as the most economically damaging plant parasitic nematodes based on yield losses and control expenses (Jones et al., 2013). As part of an international effort to phase out environmentally toxic nematicides (Zasada et al., 2010), research has focused on natural enemies as safe, sustainable control options (Stirling, 2011, 2018). The bacterium *Pasteuria penetrans* in particular has attracted long‐standing interest because it is a natural, virulent parasite of *Meloidogyne* (Mankau, 1975), limiting the establishment of juvenile hosts in roots (Vagelas et al., 2012) and castrating adult females (Mankau, 1980). Unfortunately, the results of field trials have been variable (Chen et al., 1996; Trudgill et al., 2000): release of *P. penetrans* and related species does not consistently suppress nematode populations (Bissonnette et al., 2018; Tylka et al., 2015).

Local adaptation may explain this variation in the field performance of *P. penetrans*. *P. penetrans* is an obligate parasite: It reproduces only after attaching to and invading the body of its host. Therefore, we expect *P. penetrans* populations to be under strong selection to infect locally common genotypes of *Meloidogyne* (Davies et al., 1994). Moreover, studies of *P. penetrans* provide clear evidence of specificity for infection (Channer & Gowen, 1992; Stirling, 1985). In *M. arenaria*, we established that susceptibility to infection depends on the interaction of parasite source and host line, consistent with genotype‐by‐genotype specificity (Mundim & Gibson, 2022). If *P. penetrans* adapts to local hosts, then local sources of *P. penetrans* might be the best choice for consistent, effective biological control of *Meloidogyne*. We do not yet have estimates of local adaptation required to make these management decisions.

In designing our study, we took several steps to accurately estimate local adaptation of *P. penetrans* to *M. arenaria* and shed light on the evolutionary process. First, we measured local adaptation repeatedly across 4 years, because the strength and direction of local adaptation can change from year to year (Hereford, 2009; Runquist et al., 2020). Second, we measured local adaptation at two spatial scales, within and across fields. Systems vary substantially in the spatial scale at which local adaptation is detected, from individual hosts (Blanquart & Gandon, 2013; Capelle & Neema, 2005) to geographic regions (Thrall et al., 2002). Gene flow is one determinant of the spatial scale of the adaptation, because it defines the scale at which hosts diverge genetically (Gandon, 2002; Week & Bradburd, 2023). The scale of local adaptation could be quite small in our study system: juvenile *Meloidogyne* (J2s) migrate very limited distances within the soil, on the scale of centimeters (dos Santos Oliveira et al., 2020; Prot, 1976). However, the movement of nematodes by water, wind, or machinery could increase gene flow (Esquibet et al., 2019; Lehman, 1994; Plantard & Porte, 2004). Given this uncertainty, we followed best practices in measuring local adaptation across spatial scales (Penczykowski et al., 2016; Runquist et al., 2020). Third, we measured the change in parasite local adaptation within a season to account for potential coevolution, in which hosts reciprocally evolve resistance to local parasites. Adaptation of parasites to locally common host genotypes can impose negative frequency‐dependent selection, favoring rare host genotypes to which parasites are poorly adapted. This reciprocal host evolution could mask signatures of parasite local adaptation, leading to underestimates of its strength. This problem can be addressed using a time‐shift approach to isolate the signature of parasite adaptation (Blanquart et al., 2013; Koskella, 2014). Specifically, the signature of parasite local adaptation is expected to be stronger when parasites are paired with hosts from past generations, prior to reciprocal evolution of host resistance, than with hosts from current or future generations. We accordingly compared adaptation of early‐, mid‐, and late‐season parasites to local, mid‐season hosts.

The resulting study provides the most comprehensive test thus far of adaptation of biological control agents to local host genotypes. Our results do not provide strong evidence for local adaptation in this system: in 3 of 4 years, the estimated fitness of the parasite was similar on sympatric and allopatric hosts.

## 2 MATERIALS AND METHODS

To test for local adaptation of *P. penetrans*, we collected paired samples of *M. arenaria* and *P. penetrans* from six sites each year. Because the strength of local adaptation can vary with spatial scale, we collected samples from six plots within a single field in 2019 and 2020 and from six fields in 2021 and 2022. Each year, we compared the performance of *P. penetrans* when paired with sympatric and allopatric *M. arenaria* by measuring proxies for infection probability (attachment rate) and intensity (attachment load). In 2019 and 2020, we isolated the signature of parasite adaptation by comparing local adaptation of early‐, mid‐, and late‐season parasites.

### 2.1 Natural history

*Meloidogyne arenaria* is an obligate, sedentary endoparasite of a wide diversity of plant species, including peanut (*Arachis hypogaea*). Its life cycle takes approximately 4–6 weeks to complete. Upon hatching from eggs in the soil, infective juveniles (J2) migrate in search of plant roots. They invade roots via the growing tips and establish permanent feeding sites around which galls develop as the nematodes stimulate growth and replication of the surrounding plant cells. When they reach reproductive maturity, females deposit an egg mass on the surface of the root (reviewed in Escobar et al., 2015). *M. arenaria*—like other agriculturally important species of *Meloidogyne*—reproduces asexually, via mitotic parthenogenesis (Blanc‐Mathieu et al., 2017). We have limited knowledge of the genetic diversity and structure of *M. arenaria* populations. Prior studies support the potential for substantial genetic variation and adaptive potential in this species (Blanc‐Mathieu et al., 2017; Carneiro et al., 2008; Castagnone‐Sereno & Danchin, 2014; Mundim & Gibson, 2022), in keeping with very high levels of clonal diversity observed in populations of other parthenogenetic species (Fontcuberta Garcia‐Cuenca et al., 2016; Fox et al., 1996).

*Pasteuria penetrans* is a gram‐positive, endospore‐forming bacterium that naturally parasitizes *Meloidogyne* species. Infection begins when one or more endospores attach to the cuticle of a J2 as it migrates through the soil. An attached endospore germinates after the nematode establishes within a plant root: it produces a germinal tube that pierces the cuticle of the nematode, and *P. penetrans* replicates within the body cavity. The host body ultimately fills with >2 million mature endospores, which are then released into the soil upon disintegration of the root (Mankau, 1975). We focus on the attachment step of this infection cycle, because it is readily measurable, and because *P. penetrans* cannot develop and reproduce without first attaching to a J2 (Sayre & Wergin, 1977; Stirling, 1985). Attachment does not, however, guarantee eventual infection. Therefore, we treat attachment rate as a proxy for infection probability; it is measured as the proportion of nematodes with endospores attached to their cuticles. We treat attachment load as a proxy for infection intensity; it is measured as the number of endospores attached to hosts with attachment (i.e., with at least one endospore attached).

### 2.2 2019 and 2020: Within‐field collections

**FIGURE 1:**
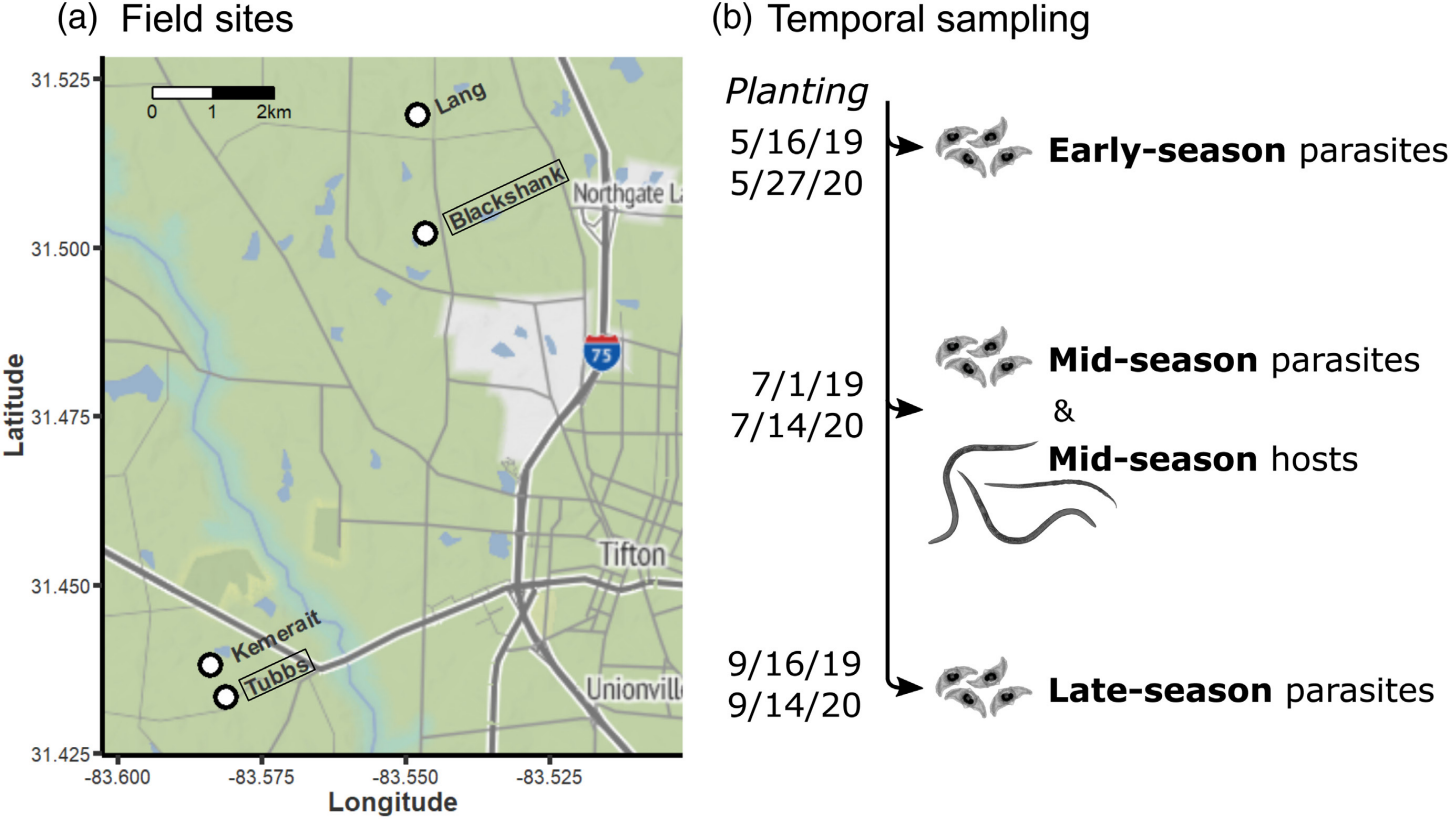
Sampling design. (a) Location of sampled fields in Tifton, GA, USA. Tubbs and Kemerait fields are part of the larger Gibbs farm. Figure S1 shows detailed layouts for boxed sites. (b) Temporal sampling scheme for 2019 and 2020 within‐field tests of local adaptation. An early‐, mid‐, and late‐season parasite sample was collected for each plot and tested against allopatric and sympatric hosts from mid‐season. 2019 and 2020 sampling dates are shown on the left.

To measure parasite local adaptation within a field, we collected host and parasite samples from six 7.3 × 6.7‐meter plots of the Tubbs field, a 0.77‐hectare peanut field at the University of Georgia Gibbs Farm in Tifton, GA, USA (Figure 1a, Figure S1a). This field has been naturally infested with *M. arenaria* and *P. penetrans* for decades (Timper et al., 2001). At the start of each season, the field is tilled, mixing soil between plots. Mundim and Gibson (2022) found that lines of *M. arenaria* from this field varied in their susceptibility to *P. penetrans*, consistent with substantial genetic variation in the host population. Liu et al. (2019) found that *P. penetrans* from different plots in this field differed in their host specificity (i.e., in which lines of *M. arenaria* they could infect) and in how host specificity changed from year to year. This study suggested highly localized adaptation of this host–parasite interaction in spite of substantial potential for gene flow between plots. These findings motivated our decision to first test for parasite local adaptation within this field, between plots.

We captured the parasite population at multiple time points throughout the growing season: when peanuts were first planted (early‐season), at mid‐season, and just before harvest (late‐season) (Figure 1b). We obtained a representative sample of *P. penetrans* endospores by collecting six to eight soil cores (2.5 cm diameter; 15 cm deep) from the center of each plot. We homogenized soil cores from a plot and heated the sample at 60°C for 2 h. This heat treatment kills native nematodes in the soil but not *P. penetrans* endospores. We stored the bulk soil sample at 4°C until use in assays. We expected soil samples to vary in abundance of endospores (i.e., dose).

We harvested hosts from each plot at mid‐season and maintained these populations in the greenhouse until the end of the season. We harvested eggs from roots, rather than J2s from soil, to ensure that *P. penetrans* endospores were not introduced to the greenhouse cultures. Specifically, we collected roots from eight peanut plants in the center of each plot and harvested eggs from the roots using bleach extraction (Coolen & D'Herde, 1972). We washed the roots to remove soil and cut them into 2–3 cm segments. We placed the root pieces in a 0.5% NaOCl solution and shook them on a rotary shaker (160 rpm) for 2 min. We then collected eggs from the solution using a 500‐mesh (25 μm) sieve. Approximately 20,000 eggs were inoculated onto three eggplants (*Solanum melongena*, cv. Black Beauty) per plot and reared in the greenhouse under natural light cycles at an average temperature of 29°C during the day and 21°C at night. Host populations were maintained for 12–20 weeks (~3–5 generations) in the greenhouse prior to use in attachment assays. While there may have been genetic change during this time due to drift or selection under culture conditions, the absence of *P. penetrans* ensured that these cultures were not subject to parasite selection. After collection of the final parasite time point, we harvested eggs from eggplants using bleach extraction, as described above. We combined eggs from the same plot in hatching pans in a mist chamber and collected hatched J2s in water every other day for 7–10 days. We preserved J2s at 4°C until attachment assays (<10 days).

### 2.3 2021 and 2022: Across‐field collections

To measure parasite local adaptation across fields, we collected host and parasite samples from multiple fields operated by the University of Georgia in Tifton, GA, USA (Figure 1a). We selected fields that we identified as having relatively high densities of *M. arenaria* and *P. penetrans* in pre‐sampling surveys conducted a few weeks before collections. In 2021, we collected samples from six fields distributed across three farms: the Tubbs and Kemerait fields at the Gibbs Farm, one field at the Lang Farm, and three fields at the Blackshank Farm, referred to as lower field 2, lower field 5, and upper field 3. In 2022, only fields at the Blackshank Farm had sufficiently high densities of *M. arenaria* and *P. penetrans* for experiments, so we collected samples from six fields at this farm: lower fields 1–4 and upper fields 3 and 4 (Figure S1b). Though these fields are geographically close, there is likely less exchange of hosts and pathogens between fields than between plots within a field: the Blackshank fields are tilled independently, so soil is not mixed between them to the same extent as it is between plots within the Tubbs field.

We collected host and parasite samples from a single time point in August, near the end of the growing season. We took three subsamples, each containing approximately eight soil cores (15–20 cm deep) from a single bed (6 × 2 m) in each field. We homogenized soil cores from the bed, dried the bulk sample at 50°C for 3 h to kill native nematodes, and stored the sample at 4°C until use in assays.

Because we used only host and parasite samples from the same time point, we collected hosts for assays directly from the field, without a maintenance period in the greenhouse. For each field, we sampled the roots of 20–24 peanut plants randomly distributed throughout the same bed from which soil was sampled. We collected eggs using bleach extraction, and hatched eggs to obtain J2s, as described above. We preserved J2s at 4°C until attachment assays (<10 days).

### 2.4 Attachment assays

Each year, we estimated attachment rate and load of *P. penetrans* in sympatric and allopatric pairings using the following assay (Timper et al., 2001). We extracted parasite endospores from a sample by thoroughly mixing the soil and subsampling 100 cm^3^ into a 250 mL flask. We added tap water to reach a final volume of 200 mL, sealed the flask, and vigorously shook it by hand for approximately 5 s. The soil particles settled for another 5 s before we decanted 100 mL of the soil‐water suspension, containing endospores, into another 250 mL flask. This established one replicate flask for that parasite sample. We collected hosts of a given sample in water as described above and counted the number of J2s in aliquots to determine the concentration. We then added a volume containing ~1000 J2s to the flask of endospores. We shook flasks on a rotary shaker at 160 rpm for 24 h. We extracted nematodes using centrifugal flotation (Caveness & Jensen, 1955; Jenkins, 1964) and counted the number of endospores attached to 30–50 nematodes per flask under 400× magnification on an inverted microscope.

In 2019 and 2020, we defined a pairing as sympatric if hosts and parasites came from the same plot in the Tubbs field, and as allopatric if they came from different plots. Each mid‐season host sample was paired with early‐, mid‐, and late‐season parasite samples from each plot, with one replicate flask per pairing. This resulted in 108 flasks (6 host plots × 6 parasite plots × 3 time points) per year (Table S1a). In 2021 and 2022, we defined a pairing as sympatric if hosts and parasites came from the same field, and as allopatric if they came from different fields. In 2021, each of our six host samples was paired with its sympatric parasite sample and two to three allopatric parasite samples. Depending on the availability of J2s, we had two or three replicate flasks per pairing. This resulted in 59 flasks (Table S1b). In 2022, each of our host samples was paired with each parasite sample, with three replicate flasks per pairing. This resulted in 108 flasks (6 host fields × 6 parasite fields × 3 replicate flasks) (Table S1c).

### 2.5 Statistical analyses

All analyses were conducted in R (v. 4.1.0) (R Core Team, 2021) and RStudio (v. 1.4.1717) (RStudio Team, 2021), using the package “tidyverse” (Wickham et al., 2019) to manipulate the data and “ggplot” and “ggmap” to generate figures (Kahle & Wickham, 2013; Wickham, 2016). We built generalized linear mixed effects models using the packages “lme4” (Bates et al., 2015) and “glmmTMB” (Brooks et al., 2017). We used DHARMa (Hartig, 2022) to check residuals and “car” (Fox & Weisberg, 2019) and ‘bbmle’ (Bolker & R. D. C. Team, 2022) to evaluate results.

Our primary goal was to estimate the effect of sympatry on variation in two proxies for *P. penetrans* fitness, attachment rate and load. For attachment rate, we fit generalized linear mixed models with a binomial distribution to the number of nematodes with and without endospores attached per replicate flask. We included a unique id for replicate flask as a random effect to correct for overdispersion. For attachment load, we excluded hosts with no endospores attached and fit generalized linear mixed models with a zero‐truncated negative binomial distribution (truncated_nbinom2 in glmmTMB) to the number of endospores per host. Comparison of residuals indicated that this distribution provided a better fit to our data than Poisson and alternative negative binomial distributions. We included flask as a random effect to account for non‐independence of nematodes from the same replicate flask. We analyzed each year separately, because the strength and direction of local adaptation can vary substantially between years (Hereford, 2009).

All models measuring local adaptation shared a common set of predictors. We first included fixed effects for host source and parasite source. We then added a fixed effect for sympatry, set to 1 for sympatric combinations of sources and 0 for allopatric combinations. This term addresses the residual variability that remains after accounting for overall effects of host and parasite source (e.g., intrinsic variation in host resistance and parasite infectivity). We would conclude that *P. penetrans* is locally adapted if there is a significant effect of sympatry, with attachment rate and/or load of *P. penetrans* higher in sympatric relative to allopatric combinations of sources. Blanquart et al. (2013) demonstrated that this “sympatric vs. allopatric” test is the most powerful and direct approach to measuring local adaptation. They also found that specifying host and parasite source as fixed effects, rather than as random effects, reduced the rate of false positives. We thus report models specifying them as fixed effects, though our results were qualitatively unchanged when we specified them as random effects.

We added additional terms to these local adaptation models based on the design of the experiment in any given year. In 2019 and 2020, we paired hosts with parasites from early‐, mid‐, and late‐season time points. Therefore, we included parasite time point and an interaction of time point and sympatry as fixed effects in 2019 and 2020 models. The interaction measures the degree to which the effect of sympatry changes with parasite time point. We predicted an increase in the effect of sympatry over the course of the season: if local parasites and hosts are reciprocally adapting to one another, then parasites sampled at the end of the season should perform relatively well on sympatric mid‐season hosts, because these hosts from the “recent past” should not yet have evolved resistance to locally adapted late‐season parasites. In contrast, parasites sampled at the start of the season should perform relatively poorly on sympatric mid‐season hosts, because these hosts from the “near future” would have evolved to resist them (Koskella, 2014) (Figure S2). In 2021 and 2022, we spread assays across three time blocks, so we added block as a fixed effect in those models.

In addition to testing for local adaptation, we took advantage of the increased replication in 2022 to test whether parasite fitness varies with the interaction of host and parasite source. This interaction term addresses the residual variation in parasite fitness on different host sources that remains after accounting for main effects of host and parasite source. A significant interaction effect would indicate that a parasite source performs better, or worse, on a given host source than expected from the additive effects of the host and parasite source. This result would be consistent with prior evidence of genotype‐by‐genotype interactions in this system (Mundim & Gibson, 2022). This analysis differs from the local adaptation analyses above, which measure the specific component of this interaction effect that is attributable to sympatry. We fit models as described above, including block, host source, parasite source, and the interaction of host and parasite sources as fixed effects. We did not evaluate this interaction in 2019–2021 because the replication scheme in those years was not conducive.

## 3 RESULTS

We tested for local adaptation of *P. penetrans* to *M. arenaria* across 4 years, varying the scale from within a single peanut field to across multiple fields. We evaluated local adaptation using two metrics, the probability of infection, measured as attachment rate, and the intensity of infection, measured as attachment load.

### 3.1 Tests of local adaptation within a field

**FIGURE 2:**
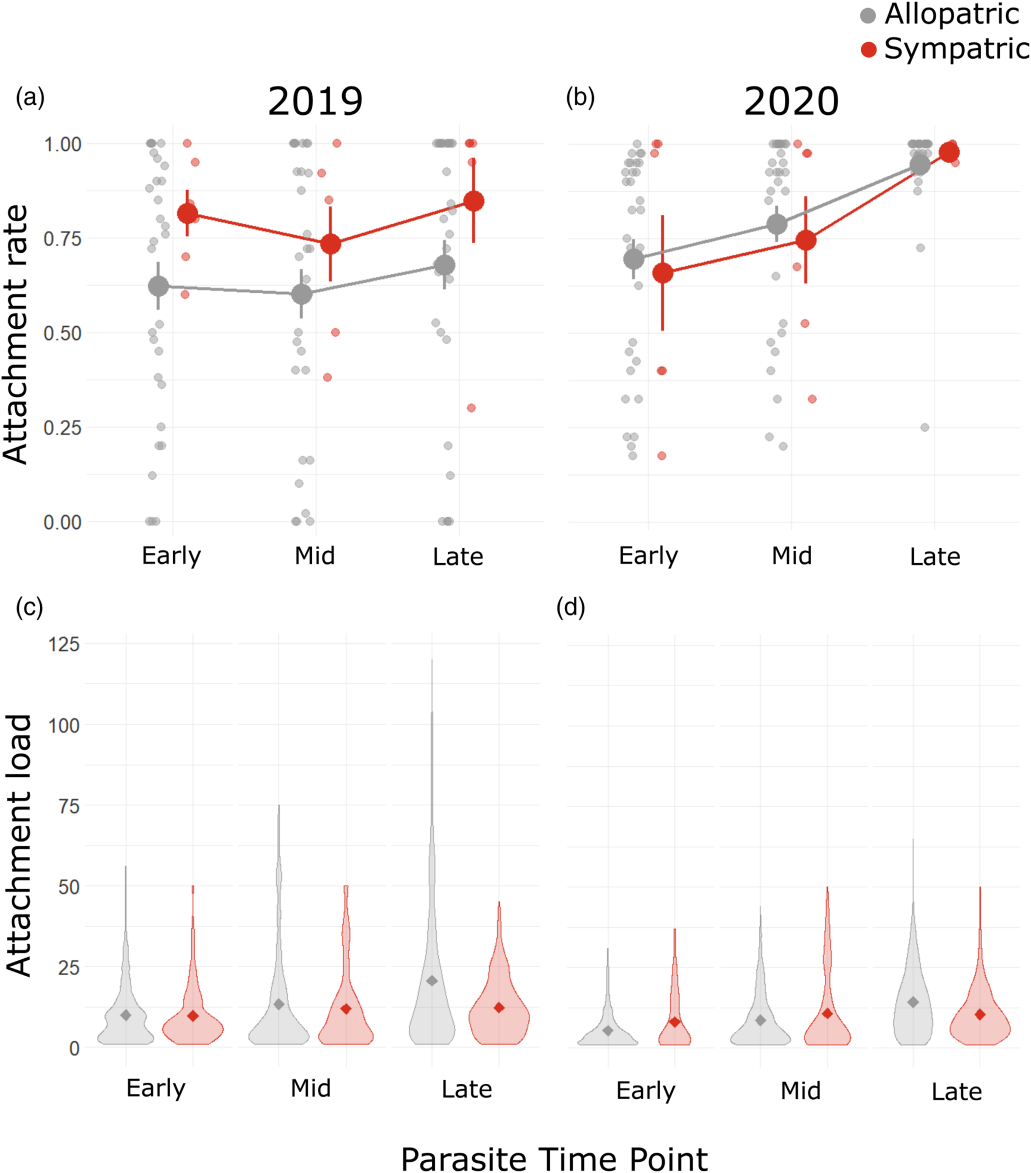
Results of within‐field tests of local adaptation. We estimated infection probability (a, b: attachment rate) and intensity (c, d: attachment load) of early‐, mid‐, and late‐season parasites in 2019 (a, c) and 2020 (b, d) in sympatry (red) and allopatry (gray). (a, b) Large points show means and standard errors; small points show individual pairings. (c, d) Violin plots show the distribution of endospores attached to individual hosts (excluding hosts with none attached), and diamonds show the mean number attached.

In 2019, attachment rate was 28% higher in sympatric relative to allopatric pairings, consistent with adaptation of parasites to local hosts (Table 1; *z* = 2.24, *p* = 0.02). However, sympatric and allopatric pairings did not differ in attachment rate in 2020, nor in attachment load in either year (Figure 2, Figure S3; Tables S2–S5). The effect of sympatry did not change with parasite time point (Tables S2 and S4: best models exclude interaction).

**TABLE 1:** Mean and standard error of attachment rate and load for sympatric and allopatric combinations in each year. Bolded values indicate a significant difference between sympatry and allopatry.

Year^a^	Combination^b^	Attachment rate^c^, %	Attachment load^d^, *endospores*
*Within a field*: 2019	Sympatric	**79.0 ± 1.40**	11.3 ± 0.39
	Allopatric	**61.8 ± 0.75**	14.8 ± 0.33
2020	Sympatric	79.4 ± 1.51	9.86 ± 0.39
	Allopatric	80.4 ± 0.67	9.78 ± 0.17
*Across fields*: 2021	Sympatric	67.8 ± 1.91	6.11 ± 0.26
	Allopatric	79.5 ± 1.01	6.49 ± 0.22
2022	Sympatric	51.9 ± 2.17	5.04 ± 0.30
	Allopatric	53.2 ± 0.97	6.60 ± 0.23

^a^ In 2019 and 2020, means reflect averages of early, mid, and late time points.

^b^ Sympatric refers to pairings of host and parasite from the same plot (2019 and 2020) or the same field (2021 and 2022). Allopatric refers to pairings of host and parasite from different plots or fields.

^c^ Proportion of hosts with endospores attached.

^d^ Number of endospores attached to hosts with attachment.

Attachment rate and load tended to increase substantially over the course of the season (Figure 2; Tables S2–S5). In 2019, attachment rate of early‐season parasites was similar to that of late‐season parasites. However, in 2020, the attachment rate of late‐season parasites was 38% greater than that of early‐season parasites (68.9 ± 1.22% vs. 94.9 ± 0.59%; *z* = 7.43, *p* < 0.001). In both years, the attachment load of late‐season parasites was approximately double that of early‐season parasites (2019: 9.91 ± 0.28 vs. 18.9 ± 0.58 endospores per J2; *z* = 3.02, *p* < 0.01; 2020: 5.82 ± 0.19 vs. 13.5 ± 0.27 endospores per J2; *z* = 10.27 *p* < 0.001).

In both years, host sources differed substantially from one another in attachment rate and load, consistent with variation in their susceptibility to infection (ΔAIC ≥20.0). Parasite sources also differed from one another, consistent with intrinsic variation in infectivity and/or endospore dose (ΔAIC ≥26.8) (Figure S3, Tables S2–S5).

### 3.2 Tests of local adaptation across fields

**FIGURE 3:**
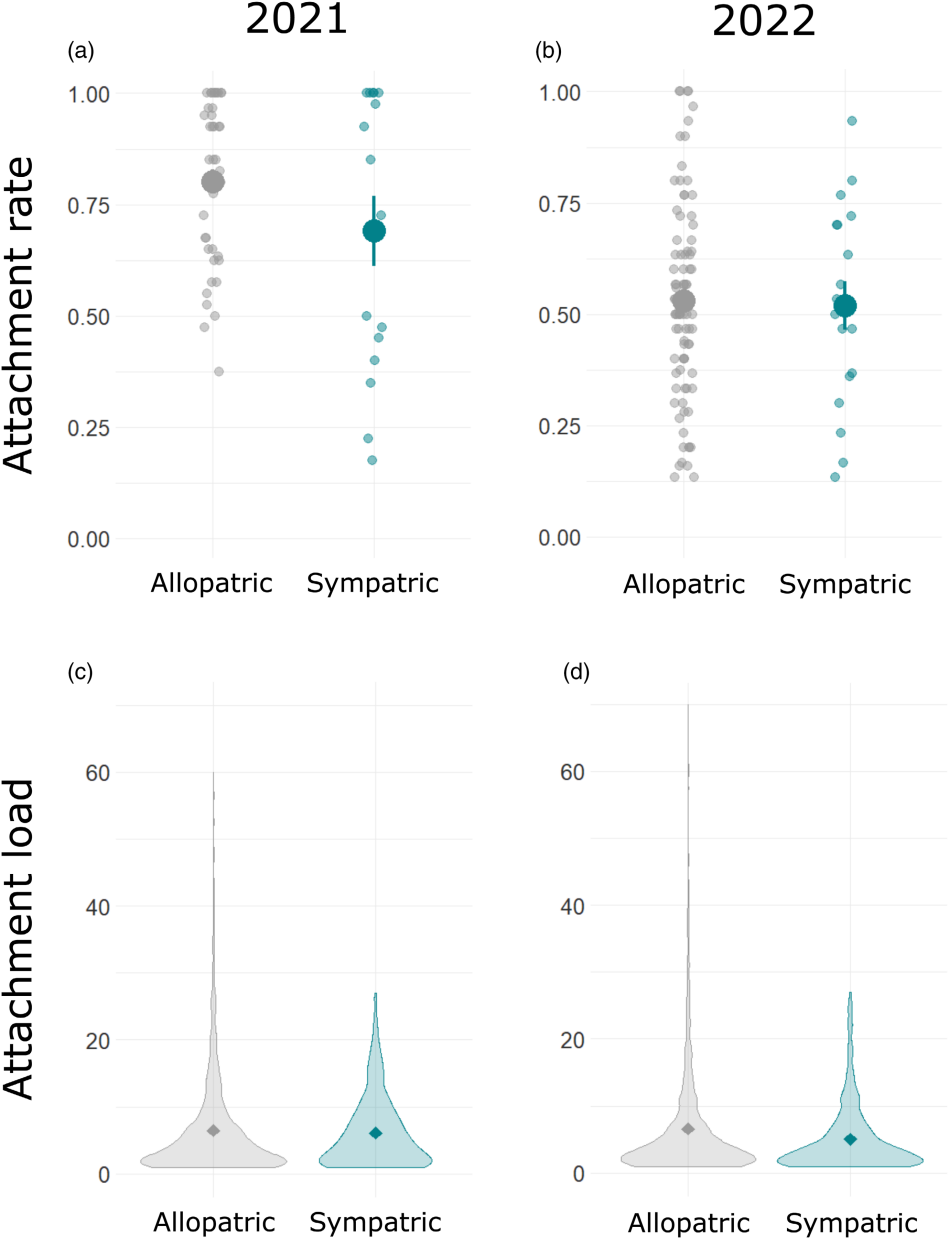
Results of across‐field tests of local adaptation. We estimated infection probability (a, b: attachment rate) and intensity (c, d: attachment load) in 2021 (a, c) and 2022 (b, d) in sympatry (blue) and allopatry (gray). Data presented as in Figure 2.

Sympatric and allopatric pairings did not differ in attachment rate or load in either year, indicating that parasites were not better at attacking hosts from their local field (Table 1, Figure 3). As in the within‐field tests, we found that parasite sources differed substantially from one another in mean attachment rate and load (ΔAIC ≥26.1). Host sources differed substantially from one another in 2021 (ΔAIC ≥17.0), but they differed only marginally in 2022 (ΔAIC ≤2.2) (Figure S4; Tables S6–S9). To follow‐up on this finding, we took advantage of the increased replication in 2022 to determine whether attachment rate and load depended upon the interaction of host and parasite source field, irrespective of sympatry. We found that neither attachment rate nor load showed a significant interaction (Table S10).

## 4 DISCUSSION

Our findings are not consistent with the hypothesis that *P. penetrans* adapts to infect locally common genotypes of *M. arenaria*. In 3 of 4 years, parasites had similar estimates of infection probability and intensity on sympatric and allopatric hosts (Table 1, Figures 2 and 3). The exception was 2019, when we found that parasites had higher infection probability when paired with hosts from the same plot relative to hosts from different plots in the same field (Figure 2a). We discuss hypotheses to explain these outcomes and their implications for management.

### 4.1 Hypotheses

The prediction of local adaptation rests on the assumption that there is some specificity for infection, such that, as parasites adapt to infect one group of hosts, they do not gain the ability to infect genetically distinct groups of hosts (Gandon, 2002). Data on *P. penetrans* provide strong evidence of specificity for infection (Channer & Gowen, 1992; Mundim & Gibson, 2022; Stirling, 1985). There have not, however, been serial passage experiments to directly test whether specificity evolves over the course of adaptation to distinct host genotypes. Channer and Gowen (1992) briefly reared a single isolate of *P. penetrans* on two lines of *M. incognita* and a mixed population of *M. incognita* and *M. javanica*. They saw mixed evidence for increases in attachment specific to the rearing host. Our results call for a longer serial passaging experiment, initiated with a genetically diverse population of *P. penetrans*, to establish whether the pattern of specificity in this system indeed supports the potential for local adaptation.

Another explanation for our results is that local adaptation manifests at larger or smaller spatial scales than we tested at. First, populations of *M. arenaria* may diverge genetically only at larger spatial scales, across regions or continents. Our largest scale tests used fields within the same county and in some cases within the same farm (Figure 1a, Figure S1b). Though population genetic data for *Meloidogyne* species are sparse (Koutsovoulos et al., 2018; Montarry et al., 2021), studies of the ecologically similar cyst nematodes *Heterodera* and *Globodera* find that gene flow can be extensive between nearby fields, and even between fields in a production region (Alenda et al., 2014; Esquibet et al., 2019; Picard et al., 2004; Plantard & Porte, 2004). Field populations of *M. arenaria* might similarly show limited genetic differentiation. This would explain why attachment in 2022 did not depend upon the interaction of host and parasite field. Mundim and Gibson (2022) found that attachment varies substantially with the interaction of parasite source and clonal host line. The absence of an interaction in 2022 suggests limited differentiation between bulk field samples of host and/or parasite, possibly reflecting extensive genetic variation that is shared between fields. However, this explanation would not account for our observation of local adaptation within a field in 2019 (Figure 2a), nor for the substantial divergence observed among host sources in other years (Figures S3 and S4).

Alternatively, the scale of local adaptation may be smaller. A study of the related *Pasteuria ramosa* also did not find clear evidence of local adaptation to its host *Daphnia magna*, even when sampling from distant ponds in England and Russia (Ebert et al., 1998). This is in spite of the fact that the *P. ramosa‐D. magna* system shows the requisite genetic specificity (Carius et al., 2001; Luijckx et al., 2013). The study found substantial genetic variation in infection traits and loci within *D. magna* populations, suggesting that parasite local adaptation is impeded by substantial shared variation in resistance loci among host populations (Bourgeois et al., 2021; Ebert et al., 1998). Ebert et al. (1998) proposed that differentiation may manifest at a smaller scale, within a pond: *D. magna* clones can be spatially structured within a pond, which could favor different *P. ramosa* lineages in different pockets of the population.

These arguments resonate with our findings for *P. penetrans*. Cyst nematodes in the genus *Heterodera* and *Globodera* often show reduced diversity and significant substructure at the level of individual plants, suggesting that restricted dispersal during the growing season results in lineages proliferating for multiple generations within the root zone of a single plant (Montarry et al., 2015). We found substantial phenotypic variation in *P. penetrans* susceptibility among isofemale lines of *M. arenaria* from a single field, suggesting abundant genetic variation within populations (Mundim & Gibson, 2022). Based on results from the cyst nematodes, this variation may be strongly structured within a field, such that distinct *M. arenaria* clones dominate the rhizosphere of individual plants. This structure could favor different *P. penetrans* lineages in different spots in a field and argues for testing parasite local adaptation at the level of individual root zones. This explanation is consistent with our sole detection of parasite local adaptation when we sampled narrowly within individual plots. Population genetic data for *M. arenaria* are needed to further evaluate these spatial hypotheses.

A final consideration is that we limited our measurement of parasite performance to the attachment stage. Parasites might also adapt to local hosts in terms of replication or virulence (Hufbauer & Roderick, 2005). In their test of *P. ramosa* local adaptation, Ebert et al. (1998) measured infection rate, endospore production, and host fitness in three sympatric and six allopatric combinations. Virulence of *P. ramosa* varied little—most infected hosts were castrated by infection. Of infections that successfully resulted in endospore production, parasites did have slightly higher endospore numbers and a faster rate of endospore production on their sympatric hosts when compared to allopatric parasites on that same host. However, this signature of local adaptation disappeared once all infections, successful or not, were accounted for. This was because sympatric and allopatric pairings did not differ in infection rate, which Ebert et al. (1998) deemed the most important trait for *P. ramosa*. The *P. ramosa*‐*D. magna* interaction bears remarkable similarity to the *P. penetrans*‐*Meloidogyne* interaction in the progression of infection (Schmidt et al., 2008). As in *P. ramosa*, attachment is the first step of infection for *P. penetrans* and is required for eventual infection. We accordingly treat attachment rate as the primary determinant of infection probability and thus as a critical trait for *P. penetrans* fitness and control potential. Nonetheless, our data do not rule out local adaptation in other *P. penetrans* traits.

### 4.2 Reciprocal adaptation

We did not detect a signature of reciprocal adaptation in 2019 or 2020 (Figure 2). The strong selection and genetic specificity of this interaction raise the potential for reciprocal adaptation via negative frequency‐dependent selection (Channer & Gowen, 1992; Liu et al., 2019). If hosts simultaneously evolve to counter locally adapting parasites, we expected parasite fitness to increase through time on sympatric hosts. Our results did not support this prediction, even in 2019 when mean parasite fitness was higher on sympatric than on allopatric hosts. Mean attachment rate and load did tend to increase over the course of the season, but this was true for both sympatric and allopatric combinations, suggesting a general gain in endospore dose or infectivity rather than local coevolution. A season may provide insufficient time to detect signatures of reciprocal adaptation; we estimate that hosts and parasites go through no more than six generations per summer. Our prediction may instead be upheld if comparisons were conducted across years. Indeed, in the *P. ramosa*‐*D. magna* system, a time‐shift experiment spanning 2–4 years found signatures consistent with coevolution via negative frequency‐dependent selection (Decaestecker et al., 2007).

Our results are also not consistent with host local adaptation for attachment susceptibility (Lemoine et al., 2012). This would manifest as lower attachment of *P. penetrans* to sympatric relative to allopatric *M. arenaria*. In no case did we see significant support for this outcome (Figures 2 and 3). In a multiyear study in microplots and paired field plots, Trudgill et al. (2000) found large increases in *P. penetrans* attachment and *Meloidogyne* control after augmenting the local population of *P. penetrans* with endospores from a foreign population. These results raised the possibility that hosts were adapted to resist local *P. penetrans*. Our results argue against this interpretation; their findings could alternately be explained by the acceleration of parasite evolution by gene flow.

### 4.3 Implications for management

Biological control with *P. penetrans* is constrained by its genotype‐by‐genotype specificity for infection of *Meloidogyne* hosts (Stirling, 1985; Trudgill et al., 2000). Our findings do not support the use of local parasites as a solution to this constraint. Though we cannot rule out local adaptation in other aspects of *P. penetrans* fitness, we do not find substantive differences between local and foreign parasites in their potential to infect. Both Channer and Gowen (1992) and Mundim and Gibson (2022) markedly increased attachment rate by increasing the number of *P. penetrans* isolates used in exposures (see also Tzortzakakis & Gowen, 1994). This increased potential for infection should correspond to increased probability of the establishment and spread of *P. penetrans* following release. Therefore, genetic diversification of *P. penetrans* remains the best supported solution to the constraint posed by genotype‐by‐genotype specificity in this biological control system.

## CONFLICT OF INTEREST STATEMENT

The authors declare no conflict of interest.

## Supporting information


Data S1.

## Data Availability

Data for this study are available at: https://doi.org/10.5061/dryad.00000009q.
