# Supplementary material for: Do biological control agents adapt to local pest genotypes? A multiyear test across geographic scales
Source: Evol Appl. 2024 Apr 11;17(4):e13682. doi: 10.1111/eva.13682 (PMC11009426; doi:10.1111/eva.13682)
Supplement: Supplementary file 1 — Data S1. [file EVA-17-e13682-s001.pdf]

Supplemental Material for

Do biological control agents adapt to local pest genotypes?
A multi-year test across geographic scales

I. SUPPLEMENTAL FIGURES

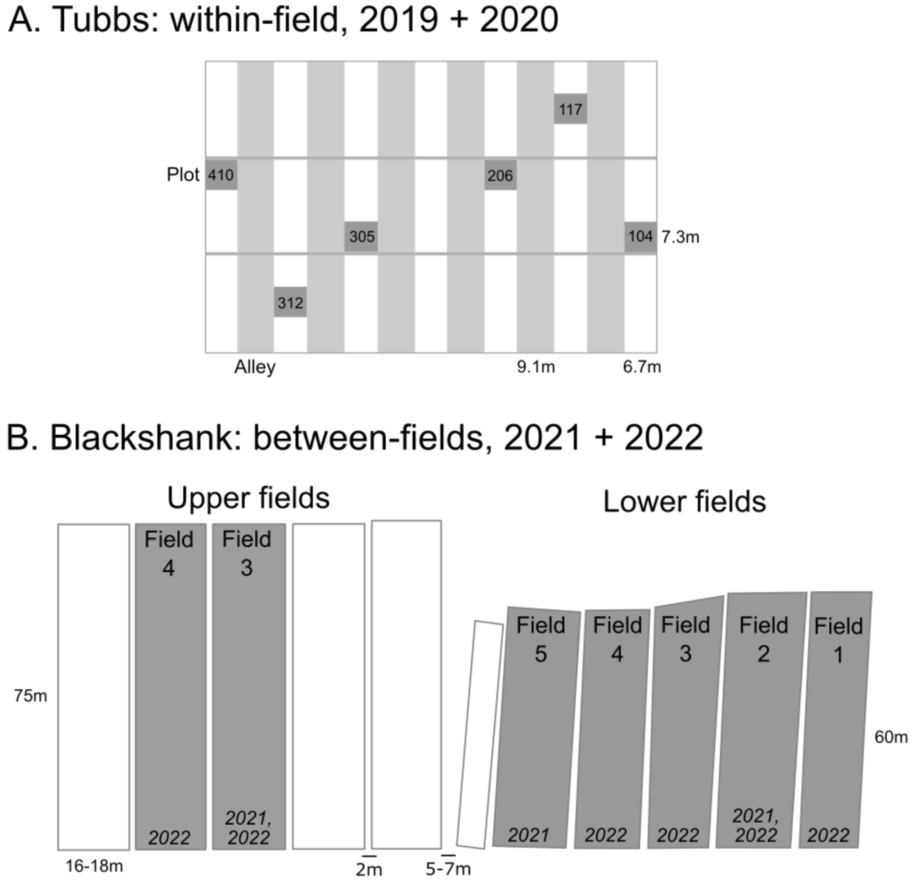

Figure S1. Field layouts. (A) Layout of Tubbs field used for within-field sampling in 2019 and 2020. Numbered boxes with dark gray shading indicate the six sampled plots. White columns are divided into nine plots each (detail not shown), and light gray columns are alleys between plots where peanuts are not planted. The field is tilled from left to right. (B) Layout of fields sampled at the Blackshank Farm. Labeled fields with dark gray shading were sampled for this study, with year(s) of sampling indicated. (A) and (B) drawn to scale.

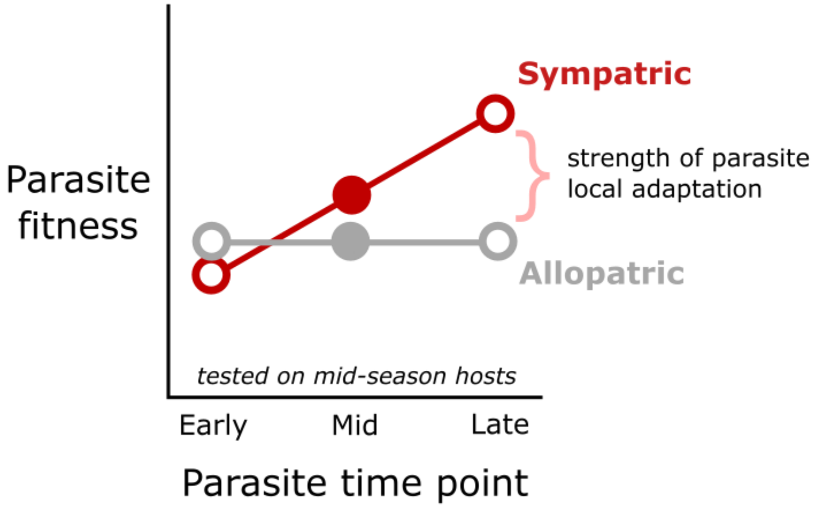

Figure S2. Schematic of prediction for local adaptation through time. In 2019 and 2020, we tested parasites collected at early-, mid-, and late-season time points on mid-season hosts. We expected that, if host and parasite are reciprocally adapting, the strength of parasite local adaptation (the difference between sympatric and allopatric fitness) would increase over the course of a season. Specifically, we predicted no mean change through time for parasite fitness in allopatry, and an increase through time in sympatry. Parasites sampled late in the season should perform relatively well on sympatric mid-season hosts, because these hosts from the "recent past" should not have evolved resistance to locally adapted late-season parasites. In contrast, parasites sampled early in the season should perform relatively poorly on sympatric mid-season hosts, because these hosts from the "near future" would have evolved to resist local parasites from the past.

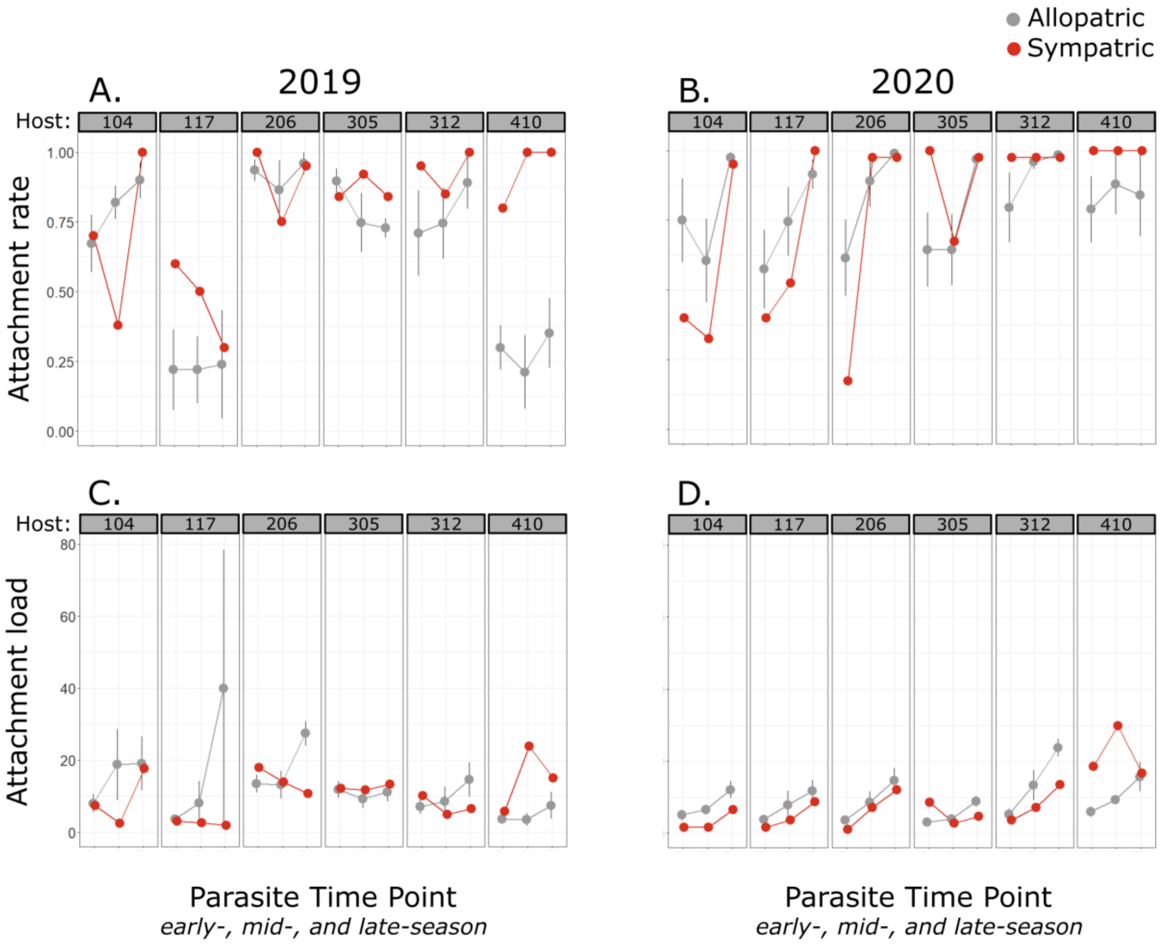

Figure S3: Results of within-field tests of local adaptation, by host source. Attachment rate (A,B) and load (C,D) are shown for allopatric (gray) and sympatric (red) parasites on each host source plot (panels). This figure thus compares the success of local and foreign parasites on a given host source. Parasites were tested from early-, mid-, and late-season time points in 2019 (A,C) and 2020 (B,D). Points show mean and standard error; error estimates are absent for sympatric combinations because we tested a single sympatric replicate per host source per time point.

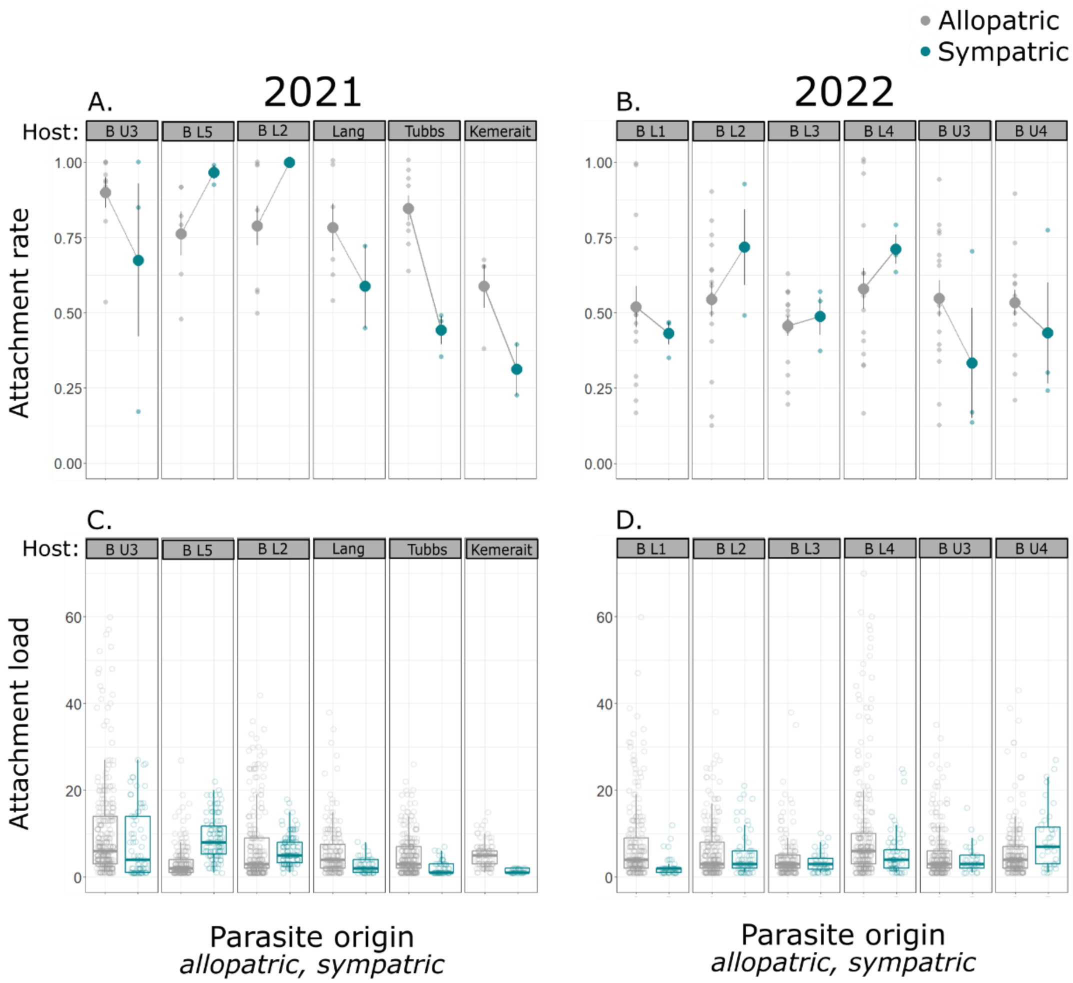

Figure S4: Results of across field tests of local adaptation, by host source. Attachment rate (A,B) and load (C,D) are shown for allopatric (gray) and sympatric (blue) parasites on each host source field (panels). Parasites were tested from a single late-season time point in 2021 (A,C) and 2022 (B,D). (A,B) Attachment rate is shown as in Figs 2 and 3. (C,D) Box plots show the distribution of parasite endospores attached to hosts (excluding hosts with none attached), with open circles depicting load for individual hosts.

II. SUPPLEMENTAL TABLES

Table S1: Crossing designs for local adaptation tests. Sympatric pairings shown in red for (a) 2019 and 2020 and in blue for (b) 2021 and (c) 2022. Cells indicate the number of replicate flasks per pairing. In 2019 and 2020, we used one replicate flask for each of three parasite samples (early, mid, and late) per pairing.

a. *2019* and *2020*

	PARASITE					
HOST	**104**	**117**	**206**	**305**	**312**	**410**
**104**	**1x3**	1x3	1x3	1x3	1x3	1x3
**117**	1x3	**1x3**	1x3	1x3	1x3	1x3
**206**	1x3	1x3	**1x3**	1x3	1x3	1x3
**305**	1x3	1x3	1x3	**1x3**	1x3	1x3
**312**	1x3	1x3	1x3	1x3	**1x3**	1x3
**410**	1x3	1x3	1x3	1x3	1x3	**1x3**

b. *2021*

	PARASITE					
HOST	**B-U3**	**B-L5**	**B-L2**	**L**	**G-T**	**G-K**
**B-U3**	**3**	3	3		3	
**B-L5**		**3**	3		3	
**B-L2**		3	**3**	3	3	
**L**	2	2	2	**2**		
**G-T**	3	3	3		**3**	
**G-K**		2	2			**2**

c. *2022*

	PARASITE					
HOST	**B-L1**	**B-L2**	**B-L3**	**B-L4**	**B-U3**	**B-U4**
**B-L1**	**3**	3	3	3	3	3
**B-L2**	3	**3**	3	3	3	3
**B-L3**	3	3	**3**	3	3	3
**B-L4**	3	3	3	**3**	3	3
**B-U3**	3	3	3	3	**3**	3
**B-U4**	3	3	3	3	3	**3**

Table S2: Model competition for variation in attachment rate in (a) 2019 and (b) 2020

a. *2019*

Full model: (Number of hosts without endospores, number of hosts with) ~ Host + Parasite + Sympatry + Time + Sympatry:Time + (1|ReplicateID)

Model Terms								
Host	*Parasite*	*Sympatry*	*Time*	*Sympatry*Time*	**Df**	**AIC**	**ΔAIC**	**Weight**
X	X	X	X		15	662.6	0.0	0.44
X	X	X			13	663.0	0.4	0.37
X	X		X		14	665.6	3.0	0.10
X	X	X	X	X	17	665.9	3.3	0.09
X		X	X	X	12	699.0	36.4	0.00
	X	X	X	X	12	736.4	73.8	0.00

b. *2020*

Full model: (Number of hosts without endospores, number of hosts with) ~ Host + Parasite + Sympatry + Time + Sympatry:Time + (1|ReplicateID)

Model Terms								
Host	*Parasite*	*Sympatry*	*Time*	*Sympatry*Time*	**Df**	**AIC**	**ΔAIC**	**Weight**
X	X		X		14	546.1	0.0	0.68
X	X	X	X		15	548.0	1.9	0.27
X	X	X	X	X	17	551.4	5.2	0.05
	X	X	X	X	12	566.1	20.0	0.00
X		X	X	X	12	592.9	46.7	0.00
X	X	X			13	594.8	48.6	0.00

Table S3: Coefficients of key models for variation in attachment rate in (a) 2019 and (b) 2020. Host and parasite levels indicate names of plots within the Tubbs field, with plot 104 as reference.

a. *2019*

Best model: (Number of hosts without endospores, number of hosts with) ~ Host + Parasite + Sympatry + Time + (1|ReplicateID)

Predictor	Level	Coefficient ± SE	z value	p value
Intercept		2.61 ± 0.77	3.41	<0.001
**Sympatric**	**Yes**	**1.25 ± 0.55**	**2.24**	**0.025**
Time point	Past	-0.85 ± 0.52	-1.64	0.102
	Present	-1.02 ± 0.52	-1.96	0.050
Host	117	-4.59 ± 0.75	-6.164	<0.001
	206	1.97 ± 0.76	2.60	0.009
	305	-0.11 ± 0.69	-0.16	0.871
	312	0.81 ± 0.72	1.12	0.261
	410	-2.72 ± 0.70	-3.89	<0.001
Parasite	117	0.25 ± 0.69	0.37	0.714
	206	-2.02 ± 0.71	-2.86	0.004
	305	-0.94 ± 0.71	-1.32	0.188
	312	-0.38 ± 0.71	-0.54	0.587
	410	3.00 ± 0.78	3.83	<0.001

b. *2020*

2nd best model: (Number of hosts without endospores, number of hosts with) ~ Host + Parasite + Sympatry + Time + (1|ReplicateID)

Predictor	Level	Coefficient ± SE	z value	p value
Intercept		2.56 ± 0.57	4.53	<0.001
**Sympatric**	**Yes**	**0.16 ± 0.43**	**0.38**	**0.708**
Time point	Past	-3.07 ± 0.41	-7.43	<0.001
	Present	-2.18 ± 0.41	-5.25	<0.001
Host	117	-0.10 ± 0.51	-0.21	0.838
	206	0.82 ± 0.53	1.56	0.119
	305	0.07 ± 0.51	0.14	0.892
	312	2.11 ± 0.56	3.74	<0.001
	410	1.54 ± 0.55	2.81	0.005
Parasite	117	1.38 ± 0.53	2.62	0.009
	206	-0.89 ± 0.49	-1.81	0.070
	305	1.95 ± 0.54	3.64	<0.001
	312	1.32 ± 0.51	2.57	0.010
	410	3.04 ± 0.59	5.14	<0.001

Table S4: Models for variation in attachment load in (a) 2019 and (b) 2020

a. *2019*

Full model: Spore number ~ Host + Parasite + Sympatry + Time + Sympatry:Time + (1|ReplicateID)

Model Terms								
Host	*Parasite*	*Sympatry*	*Time*	*Sympatry*Time*	**Df**	**AIC**	**ΔAIC**	**Weight**
X	X		X		15	21057.3	0.0	0.65
X	X	X	X		16	21059.2	1.8	0.26
X	X	X	X	X	18	21061.4	4.0	0.09
X	X	X			14	21067.5	10.2	0.00
X		X	X	X	13	21084.1	26.8	0.00
	X	X	X	X	13	21088.5	31.1	0.00

b. *2020*

Full model: Spore number ~ Host + Parasite + Sympatry + Time + Sympatry:Time + (1|ReplicateID)

Model Terms								
Host	*Parasite*	*Sympatry*	*Time*	*Sympatry*Time*	**Df**	**AIC**	**ΔAIC**	**Weight**
X	X	X	X		16	19678.66	0.0	0.54
X	X		X		15	19679.38	0.7	0.38
X	X	X	X	X	18	19682.57	3.9	0.08
	X	X	X	X	13	19706.95	28.3	0.00
X		X	X	X	13	19720.75	42.1	0.00
X	X	X			14	19751.23	72.6	0.00

Table S5: Coefficients of key models for variation in attachment load in (a) 2019 and (b) 2020. Host and parasite levels indicate names of plots within the Tubbs field, with plot 104 as reference.

a. *2019*

2nd best model: Spores ~ Host + Parasite + Sympatry + Time + (1|ReplicateID)

Predictor	Level	Coefficient ± SE	z value	p value
Intercept		1.96 ± 0.26	7.64	<0.001
**Sympatric**	**Yes**	**0.07 ± 0.19**	**0.39**	**0.698**
Time point	Past	-0.54 ± 0.18	-3.02	0.003
	Present	-0.59 ± 0.18	-3.24	0.001
Host	117	-1.00 ± 0.30	-3.34	0.001
	206	0.45 ± 0.24	1.88	0.060
	305	0.00 ± 0.24	0.01	0.989
	312	-0.32 ± 0.24	-1.34	0.180
	410	-0.94 ± 0.26	-3.68	<0.001
Parasite	117	0.46 ± 0.24	1.87	0.062
	206	0.55 ± 0.26	2.09	0.036
	305	0.59 ± 0.26	2.26	0.024
	312	0.96 ± 0.26	3.76	<0.001
	410	1.39 ± 0.24	5.73	<0.001

b. *2020*

Best model: Spores ~ Host + Parasite + Sympatry + Time + (1|ReplicateID)

Predictor	Level	Coefficient ± SE	z value	p value
Intercept		1.91 ± 0.20	9.75	<0.001
**Sympatric**	**Yes**	**-0.24 ± 0.14**	**-1.65**	**0.099**
Time point	Past	-1.36 ± 0.13	-10.27	<0.001
	Present	-0.69 ± 0.13	-5.36	<0.001
Host	117	-0.06 ± 0.19	-0.33	0.744
	206	0.10 ± 0.19	0.55	0.580
	305	-0.24 ± 0.19	-1.27	0.205
	312	0.61 ± 0.18	3.30	0.001
	410	0.66 ± 0.18	3.58	<0.001
Parasite	117	0.38 ± 0.19	2.05	0.040
	206	0.03 ± 0.19	0.14	0.886
	305	0.63 ± 0.19	3.38	0.001
	312	0.31 ± 0.19	1.67	0.095
	410	1.20 ± 0.19	6.47	<0.001

Table S6: Models for variation in attachment rate in (a) 2021 and (b) 2022

a. *2021*

Full model: (Number of hosts without endospores, number of hosts with) ~ Host + Parasite + Sympatry + Block + (1|ReplicateID)

Model Terms							
Host	*Parasite*	*Sympatry*	*Block*	**Df**	**AIC**	**ΔAIC**	**Weight**
X	X	X		13	326.99	0.0	0.52
X	X		X	14	327.89	0.9	0.33
X	X	X	X	15	329.57	2.6	0.14
	X	X	X	10	354.46	27.5	0.00
X		X	X	10	380.12	53.1	0.00

b. *2022*

Full model: (Number of hosts without endospores, number of hosts with) ~ Host + Parasite + Sympatry + Block + (1|ReplicateID)

Model Terms							
Host	*Parasite*	*Sympatry*	*Block*	**Df**	**AIC**	**ΔAIC**	**Weight**
X	X		X	14	662.81	0.0	0.55
	X	X	X	10	664.64	1.8	0.22
X	X	X	X	15	664.66	1.9	0.22
X	X	X		13	670.30	7.5	0.01
X		X	X	10	719.05	56.2	0.00

Table S7: Coefficients of key models for variation in attachment rate in (a) 2021 and (b) 2022. Host and parasite levels indicate field names (farm: field), with Black: Upper 3 (Blackshank Farm, upper field 3) as reference in 2021 and Black: Lower 1 as reference in 2022.

a. *2021*

Full model: (Number of hosts without endospores, number of hosts with) ~ Host + Parasite + Sympatry + Block + (1|ReplicateID)

Predictor	Level	Coefficient ± SE	z value	p value
Intercept		0.99 ± 0.63	1.59	0.113
**Sympatric**	**Yes**	**0.20 ± 0.36**	**0.56**	**0.578**
Host	Black: Lower 5	-0.31 ± 0.60	-0.52	0.604
	Black: Lower 2	1.06 ± 0.64	1.65	0.100
	Lang	-0.90 ± 0.55	-1.63	0.104
	Gibbs: Tubbs	-0.75 ± 0.53	-1.43	0.154
	Gibbs: Kemerait	-3.50 ± 0.65	-5.35	<0.001
Parasite	Black: Lower 5	2.78 ± 0.52	5.37	<0.001
	Black: Lower 2	1.76 ± 0.47	3.73	<0.001
	Lang	-1.34 ± 0.63	-2.14	0.032
	Gibbs: Tubbs	-0.42 ± 0.47	-0.91	0.366
	Gibbs: Kemerait	0.87 ± 0.96	0.90	0.369
Block	2	0.17 ± 0.46	0.36	0.716
	3	0.61 ± 0.52	1.17	0.242

b. *2022*

Full model: (Number of hosts without endospores, number of hosts with) ~ Host + Parasite + Sympatry + Block + (1|ReplicateID)

Predictor	Level	Coefficient ± SE	z value	p value
Intercept		0.07 ± 0.27	0.25	0.802
**Sympatric**	**Yes**	**-0.08 ± 0.20**	**-0.38**	**0.702**
Host	Black: Lower 2	0.25 ± 0.26	0.97	0.334
	Black: Lower 3	-0.28 ± 0.26	-1.11	0.268
	Black: Lower 4	0.47 ± 0.26	1.80	0.071
	Black: Upper 3	-0.04 ± 0.26	-0.17	0.864
	Black: Upper 4	-0.02 ± 0.26	-0.06	0.949
Parasite	Black: Lower 2	1.63 ± 0.27	6.12	<0.001
	Black: Lower 3	0.25 ± 0.25	0.99	0.322
	Black: Lower 4	0.71 ± 0.25	2.79	0.005
	Black: Upper 3	-0.57 ± 0.26	-2.23	0.026
	Black: Upper 4	0.05 ± 0.25	0.20	0.840
Block	2	-0.58 ± 0.18	-3.17	0.002
	3	-0.31 ± 0.18	-1.68	0.093

Table S8: Models for variation in attachment load in (a) 2021 and (b) 2022

a. *2021*
Full model: Spore number ~ Host + Parasite + Sympatry + Block + (1|ReplicateID)

Model Terms							
Host	*Parasite*	*Sympatry*	*Block*	**Df**	**AIC**	**ΔAIC**	**Weight**
X	X	X	X	16	8109.48	0.0	0.52
X	X		X	15	8109.71	0.2	0.47
X	X	X		14	8117.94	8.5	0.01
	X	X	X	11	8126.51	17.0	0.00
X		X	X	11	8180.94	71.5	0.00

b. *2022*
Full model: Spore number ~ Host + Parasite + Sympatry + Block + (1|ReplicateID)

Model Terms							
Host	*Parasite*	*Sympatry*	*Block*	**Df**	**AIC**	**ΔAIC**	**Weight**
X	X	X		14	8553.24	0.0	0.36
X	X	X	X	16	8553.32	0.1	0.34
X	X		X	15	8554.62	1.4	0.18
	X	X	X	11	8555.48	2.2	0.12
X		X	X	11	8579.34	26.1	0.00

Table S9: Coefficients of key models for variation in attachment load in (a) 2021 and (b) 2022. Host and parasite levels indicate field names (farm: field), with Black: Upper 3 (Blackshank Farm, upper field 3) as reference in 2021 and Black: Lower 1 as reference in 2022.

a. *2021*

Full model: Spore number ~ Host + Parasite + Sympatry + Block+ (1|ReplicateID)

Predictor	Level	Coefficient ± SE	z value	p value
Intercept		0.89 ± 0.24	3.65	<0.001
**Sympatric**	**Yes**	**0.20 ± 0.13**	**1.51**	**0.130**
Host	Black: Lower 5	-0.22 ± 0.22	-0.99	0.324
	Black: Lower 2	0.06 ± 0.22	0.29	0.770
	Lang	-081± 0.20	-3.98	<0.001
	Gibbs: Tubbs	-0.64 ± 0.20	-3.27	0.001
	Gibbs: Kemerait	-1.00 ± 0.25	-4.04	<0.001
Parasite	Black: Lower 5	1.46 ± 0.19	7.71	<0.001
	Black: Lower 2	0.66 ± 0.19	3.46	<0.001
	Lang	-0.46 ± 0.27	-1.72	0.085
	Gibbs: Tubbs	-0.09 ± 0.21	-0.46	0.649
	Gibbs: Kemerait	-1.32 ± 0.52	-2.54	0.011
Block	2	-0.10 ± 0.16	-0.61	0.544
	3	0.61 ± 0.20	3.11	0.002

b. *2022*

Full model: Spore number ~ Host + Parasite + Sympatry + Block + (1|ReplicateID)

Predictor	Level	Coefficient ± SE	z value	p value
Intercept		0.95 ± 0.21	4.46	<0.001
**Sympatric**	**Yes**	**-0.29 ± 0.16**	**-1.82**	**0.068**
Host	Black: Lower 2	0.14 ± 0.20	0.68	0.498
	Black: Lower 3	-0.26 ± 0.20	-1.28	0.202
	Black: Lower 4	0.40 ± 0.20	2.04	0.041
	Black: Upper 3	-0.06 ± 0.20	-0.29	0.774
	Black: Upper 4	0.15 ± 0.20	0.76	0.450
Parasite	Black: Lower 2	0.99 ± 0.20	5.05	<0.001
	Black: Lower 3	0.30 ± 0.20	1.49	0.137
	Black: Lower 4	0.64 ± 0.20	3.23	0.001
	Black: Upper 3	-0.12 ± 0.21	-0.56	0.574
	Black: Upper 4	0.37 ± 0.20	1.83	0.068
Block	2	-0.20 ± 0.14	-1.43	0.152
	3	0.07 ± 0.14	0.51	0.608

Table S10: Evaluating the interaction of host and parasite source field as predictors of variation in (a) attachment rate and (b) load in 2022

a. *Attachment rate*
Full model: (Number of hosts without endospores, number of hosts with) ~ Host * Parasite + Block + (1|ReplicateID)

Model Terms							
Host	*Parasite*	*Interaction*	*Block*	**Df**	**AIC**	**ΔAIC**	**Weight**
	X		X	9	662.79	0.00	0.50
X	X		X	14	662.81	0.03	0.50
X	X	X	X	39	676.66	13.88	0.00
X	X	X		37	685.17	22.38	0.00
X			X	9	717.14	54.36	0.00

b. *Attachment load*
Full model: Spore number ~ Host * Parasite + Block + (1|ReplicateID)

Model Terms							
Host	*Parasite*	*Interaction*	*Block*	**Df**	**AIC**	**ΔAIC**	**Weight**
X	X		X	15	8554.62	0.0	0.70
	X		X	10	8556.36	1.7	0.30
X	X	X	X	40	8569.51	14.9	0.00
X	X	X		38	8570.96	16.3	0.00
X			X	10	8579.69	25.1	0.00
